# Ultrasonographical Evaluation of Placenta Previa in Scarred and Unscarred Uterus in a Tertiary Care Centre

**DOI:** 10.7759/cureus.42586

**Published:** 2023-07-27

**Authors:** Lakshmi R Taye, Bharati Basumatari, Manik C Das, Putul Mahanta

**Affiliations:** 1 Radiology, Fakhruddin Ali Ahmed Medical College and Hospital, Barpeta, IND; 2 Radiology, Nalbari Medical College and Hospital, Nalbari, IND; 3 Forensic Medicine and Toxicology, Nalbari Medical College and Hospital, Nalbari, IND

**Keywords:** alcohol consumption, multiparity, ultrasonography, scarred uterus, placenta previa

## Abstract

Objectives

Placenta previa is characterised as the placenta implant in the lower uterine segment, wholly or partially covering the internal os. Uterine scars from surgical operations are a potential factor of placenta previa. The present study aims to estimate the role of ultrasound in determining the incidence of placenta previa in the scarred and unscarred uterus. Also, it aims to evaluate the types of placenta previa in the scarred and unscarred uterus.

Methods

This hospital-based, prospective, observational study was performed from September 2021 to August 2022 among patients referred to the Department of Radiology, Fakhruddin Ali Ahmed Medical College and Hospital (FAAMCH), Barpeta, Assam. Written informed consent was obtained from the subjects. Transabdominal and transvaginal ultrasonography methods were used to assess placenta previa. The data analysis was performed using Statistical Package for the Social Sciences (SPSS) version 21 (IBM Corp., Armonk, NY) considering a p-value < 0.05 as significant.

Results

Out of the 517 subjects with bleeding per vagina, 41 (7.9%) were diagnosed with placenta previa by ultrasonography. The mean maternal age was 27.80 ± 5.36 years, and the most prevalent age group was 20-24 years (31.71%). The majority (70.73%) of cases had scarred uterus. The most prevalent placental position was fundo-body anterior. Complete placenta previa was present in 26% of the total cases in the present study.

Conclusion

The incidence of placenta previa in the scarred uterus was higher than that of the unscarred uterus. The high prevalence of placenta previa in women with scarred uterus necessitates improved monitoring and management to avoid disastrous outcomes.

## Introduction

Placenta previa is the implantation of the placenta in the lower uterine segment, wholly or partially covering the internal os [1-3]. Placenta previa is distinguished by bleeding after 28 weeks of gestation. It is a significant risk factor for postpartum haemorrhage, which can result in morbidity and fatality for both the mother and the newborn [4]. Placenta previa may result in adverse foetal complications, including intrauterine growth restriction, preterm delivery, and foetal demise. It may also result in severe maternal complications like antepartum haemorrhage, maternal anaemia, shock, and surgical interventions like caesarean sections and hysterectomies [5,6]. As caesarean sections have increased, placenta previa, which affects 0.3% to 2% of third-trimester pregnancies, has become increasingly prevalent [3,4,7].

Gravidity, parity, previous caesarean delivery, uterine scars, pathology, and smoking abuse are some risk factors of placenta previa [8-11]. Uterine scars from surgical operations, such as myomectomy, dilatation and curettage, hysterotomy, and abortive surgical procedures, as well as endometritis, submucous fibroids, adenomyosis, and uterine adhesions, may all be risk factors for placenta previa.

Usually, more than the clinical history and examination is required to make a conclusive and correct diagnosis. Ultrasound imaging is essential for identifying placenta previa in both scarred and unscarred uterus. The gold standard for diagnosing placenta previa is transvaginal ultrasonography (TVS) [12].

The present study aims to estimate the role of ultrasound in determining the incidence of placenta previa in the scarred and unscarred uterus. Also, it aims to evaluate the types of placenta previa in the scarred and unscarred uterus.

## Materials and methods

This hospital-based, prospective, observational study was performed from September 2021 to August 2022. Data were obtained from patients referred to the Department of Radiology, Fakhruddin Ali Ahmed Medical College and Hospital (FAAMCH), Barpeta, Assam. Patient selection was done using a purposive sampling technique. Written and informed consent was obtained from the subjects before initiating the study.

Patients attending the emergency labour room and antenatal clinic of the Department of Obstetrics and Gynaecology being referred to the Department of Radiology, FAAMCH, who met our inclusion and exclusion criteria, were chosen as the cases for the study. A total of 517 subjects presenting with bleeding per vagina were enrolled in the study. They were assessed for placenta previa using transabdominal ultrasonography (TAS) and transvaginal ultrasonography (TVS).

This study included all pregnant women with complaints of vaginal bleeding, especially in the second half of pregnancy after 28 weeks until the onset of labour, who were willing to participate. All patients with non-obstetrical causes of vaginal bleeding and pregnant patients with bleeding disorders and coagulopathies were excluded. Those who were not willing to participate in the study were also excluded. A detailed clinical history and examination were done, and data were recorded on a pre-designed proforma.

After taking proper consent, all the cases were evaluated in the Samsung RS80A ultrasonography machine. The probes employed were curvilinear array transducers with frequencies 3.5-5 MHz for transabdominal sonography, and endovaginal transducers with frequencies 5-7.5 MHz were used for transvaginal ultrasonography. The patient was examined supine for transabdominal sonography and put in the lithotomy position for transvaginal ultrasonography.

Data analysis

The data analysis was performed using Statistical Package for the Social Sciences (SPSS) version 21 (IBM Corp., Armonk, NY). The chi-square test was used for testing between categorical variables, and differences in proportions were tested using the z-test. Differences between continuous variables were tested using Student’s t-test. A p-value < 0.05 is considered significant.

## Results

**Figure 1:**
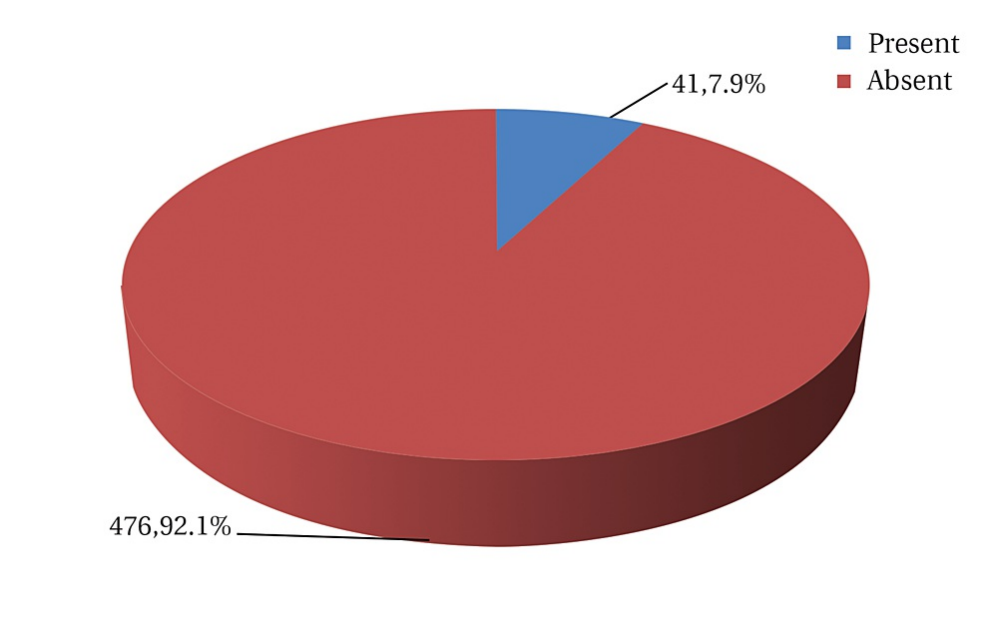
Incidence of placenta previa among the study participants

A total of 517 women presenting with bleeding in the later half of pregnancy after 28 weeks of gestation were evaluated by transabdominal and transvaginal ultrasonography to estimate the incidence of placenta previa and its types in the scarred and unscarred uterus. Out of the 517 subjects presented with bleeding per vagina, 41 were diagnosed with placenta previa by ultrasonography with an incidence rate of 7.9% (Figure 1).

The mean maternal age of the participants with placenta previa was 27.80 ± 5.36 years. It was observed that the most prevalent age group was 20-24 years (31.71%), followed by 30-34 years (29.27%) and 25-29 years (26.83%) (Table 1).

**Table 1:** Distribution of placenta previa cases according to the age

Age in years	Frequency (n = 41)	Percentage (%)
20-24	13	31.7%
25-29	11	26.8%
30-34	12	29.3%
35-39	5	12.2%

As seen from Table 2, out of 41 subjects with placenta previa, the majority (70.73%) had scarred uterus.

**Table 2:** Placenta previa in a scarred and unscarred uterus

Uterus	Frequency	Percentage (%)	p-value
Scarred	29	70.73%	0.0001
Unscarred	12	29.27%	

The distribution of placenta previa was significantly different in participants with the scarred and unscarred uterus (p < 0.05). Table 3 shows the general characteristics of the placental previa cases under study.

**Table 3:** Characteristics of the placenta previa cases # Presented as mean (±standard deviation).

Characteristics		Uterus type		p-value
		Scarred	Unscarred	
Parity	Multi	24 (82.8%)	10 (83.3%)	0.96
	Primi	5 (17.2%)	2 (16.7%)	
Previous placenta previa	No	28 (96.6%)	11 (91.7%)	0.97
	Yes	1 (3.4%)	1 (8.3%)	
Haemoglobin (in g/dl)^#^		8.4 (1.4)	8.5 (1.5)	0.09
Gestational age (GA) in weeks^#^		30.9 (2.2)	31.9 (2.6)	0.09
Smoking	No	27 (93.1%)	10 (83.3%)	0.33
	Yes	2 (6.9%)	2 (16.7%)	
Alcohol	No	24 (82.8%)	11 (91.7%)	0.54
	Yes	5 (17.2%)	1 (8.3%)	
Pain abdomen	Present	11 (37.9%)	5 (41.7%)	0.82
	Absent	18 (62.1%)	7 (58.3%)	

Majority of the placenta previa patients were multiparous. Multiparity was noted in 82.8% of cases in scarred group and 83.3% in the unscarred group. Almost 17% of women with scarred uterus were primiparous. Primiparity among scarred uterus is rare, as uterine surgical procedures like previous caesarean section or dilation and curettage (D&C) for a miscarriage, abortion, or retained placenta after delivery mainly contribute to uterus scarring. However, it is not uncommon, as endometriosis and other infection or inflammations might also cause uterine adhesions or scarring in non-pregnant women. No significant association was found between parity and placenta previa in the scarred and unscarred uterus (p > 0.05). Previous placenta previa was noted in one subject, each with a scarred and unscarred uterus. The overall mean (±SD) haemoglobin level among the placenta previa cases was 8.5 ± 1.5 g/dl. No significant difference (p > 0.05) in the mean haemoglobin level was observed between scarred and unscarred uterus cases. There was no significant difference (p > 0.05) in the mean gestational weeks (GA) between scarred and unscarred uterus with placenta previa.

Almost 93% of cases in the scarred group and 83.3% in the unscarred group were non-smokers. No significant association was found between smoking and placenta previa in the scarred or unscarred uterus (p > 0.05). Alcohol consumption was noted among 14.6% of the cases. No significant association was found between alcohol consumption and placenta previa between the two groups. Among the cases with the scarred uterus, 11 (37.9%) subjects had abdominal pain. While in the unscarred uterus group, five (41.7%) cases had abdominal pain. No significant association was observed between the presence of abdominal pain and the type of uterus among placenta previa cases (Table 3).

The most prevalent placental position was fundo-body anterior, with almost 55% in the scarred group and 67% in the unscarred group. A significant association was found between placental position and uterus type (Table 4).

**Table 4:** Placental position in placenta previa patients

Placental position	Uterus type		p-value
	Scarred (n = 29)	Unscarred (n = 12)	
Fundo-body anterior	16 (55.2%)	8( 66.7%)	0.002
Fundo-body posterior	13 (44.8%)	4 (33.3%)	

According to TAS, out of 29 patients with a scarred uterus, type I placenta previa was diagnosed in 34.5% of cases, and type II and type IV in 31.0% of patients each. While by TVS, type I, type II, and type III placenta previa constituted 31.0% of cases each in scarred uterus group. Both TAS and TVS diagnosed 33.3% of cases as type I, 25.0% of cases each as type II and type III, and 16.7% of cases as having type IV placenta previa in the unscarred group. No significant difference (p-value > 0.05) was observed between the type of placenta previa diagnosed by TAS or TVS with uterus type, as shown in Table 5.

**Table 5:** Types of placenta previa on transabdominal ultrasonography

Ultrasonography	Type of placenta previa	Uterus type		p-value
		Scarred (n = 29)	Unscarred (n = 12)	
Transabdominal (TAS)	Type I	10 (34.5%)	4 (33.3%)	0.871
	Type II	9 (31.0%)	3 (25.0%)	
	Type III	1 (3.4%)	3 (25.0%)	
	Type IV	9 (31.0%)	2 (16.7%)	
Transvaginal (TVS)	Type I	9 (31.0%)	4 (33.3%)	0.383
	Type II	9 (31.0%)	3 (25.0%)	
	Type III	2 (6.9%)	3 (25.0%)	
	Type IV	9 (31.0%)	2 (16.7%)	

## Discussion

Placenta previa represents 1/5th of antepartum bleeding affecting 0.4-0.5% of all pregnancies and is a significant cause of maternal and foetal death [7,8]. In India, placenta previa, eclampsia, and sepsis are the three major causes of maternal death [9]. Multiparty, multiple gestations, uterine surgery, advanced maternal age, dilatation and curettage, history of placenta previa, assisted reproductive technology, smoking, and other substance use, low socioeconomic status, etc. are contributory factors of placenta previa [6]. The placentation type, mainly percreta, was recently associated with severe complications [10]. A TVS is the gold standard for diagnosing placenta previa [7].

The present study’s objective was to estimate the role of ultrasound in determining the incidence of placenta previa in the scarred and unscarred uterus and to evaluate the types of placenta previa in the scarred and unscarred uterus.

Out of the 517 subjects presented with bleeding per vagina, the incidence of placenta previa in the study was 7.93%. The incidence of placenta previa in the present study population was higher than in similar studies [8,11]. In systematic reviews, the pooled prevalence of placenta previa was approximately four per 1000 births but varied worldwide [1]. A study from Mainland China from 1965 to 2015 reported the pooled overall prevalence of placenta previa among deliveries as 1.24% [12]. A recent study from India, including 6840 patients, reported the incidence of placenta previa as only 0.51% [9], somewhat lower than the current study.

In this study, the majority (58.54%) of the cases were presented before the age of 30 years. The findings agree with other studies [9,13]. In contrast to our findings, another study reported that placenta previa increased with maternal age and was the highest in women aged 35 years or older. The disagreement may be due to the low sample size of our study [14].

In the present study, the frequency of placenta previa was higher in the scarred uterus (70.73%) than in the unscarred uterus. A similar study from Pakistan reported the frequency of placenta previa in the previously scarred uterus as 67.54% [15]. Another research reported more cases with previous uterine operations in the placenta previa group than in the control group [16]. Yazdani et al. reported that 15.5% of patients with a previous history of caesarean section were diagnosed with placenta previa [17]. Akram et al. reported that 23.3% of patients with placenta previa had a history of previous caesarean section [18]. It is biologically conceivable that placenta previa and placental abruption are related to previous caesarean deliveries [19]. A uterine low-segment scar is detrimental to the placental attachment. Ligating uterine vessels after a caesarean section may raise the chance of endometrial, myometrial, or uterine lining injury, which could lead to lower placenta implantation during the subsequent pregnancy. It is hypothesised that the uterine muscle cut during abdominal delivery interferes with natural stretching and prevents or hinders the placenta from travelling away to the upper uterine segment in a subsequent pregnancy [20-23].

Placenta previa was primarily present in multiparous women, with 82.76% of cases in the scarred uterus group and 83.33% in the unscarred uterus group. Various studies have also reported that placenta previa is more common among multiparous cases [22,23]. No significant difference in the mean haemoglobin level was observed between scarred and unscarred uterus group with placenta previa (p > 0.05). In contrast to our findings, a previous study reported anterior placental location (OR: 2.48; 95% CI: 1.20-5.11) as an independent risk factor of neonatal anaemia after controlling for potential confounders [24].

Gestational age at ultrasound detection of placenta previa is a predictor of the likelihood of previa persistence [25]. In the present study, no significant variability was observed in the mean gestational weeks (GA) between scarred and unscarred uterus with placenta previa (p > 0.05).

Studies from Western countries reported cigarette smoking and cocaine abuse as contributory factors of placenta previa [26]. Also, various researchers have reported significant associations between alcohol consumption during pregnancy and placental abruption rather than placenta previa [26,27]. But in the present study, no significant association could be established between smoking and alcoholism with placenta previa in patients with a scarred or unscarred uterus. This may be because the present study was a time-bound, hospital-based, prospective observational study with a small sample size.

In our study, anterior placenta previa was observed more in scarred and unscarred uterus groups. Various studies have reported anterior previa as more common in the scarred uterus than the unscarred uterus, which contradicts our findings [28,29]. Complete placenta previa (type IV) was present in 26% of the total cases in the present study. The findings can be compared to other similar research [13,30].

Limitations

The present study was a single-centre study conducted in a rural medical college and hospital with limited resources. It could have been conducted in a few other similar tertiary care centres in the same region, which still needs to be done.

Further, the status of the patient with placenta previa type I opting for standard vaginal delivery expecting the risk of massive blood loss and the need for blood transfusion was not known as the study did not collaborate with the obstetrics and gynaecology department, which could have been done.

## Conclusions

The incidence of placenta previa in the scarred uterus was higher than that of the unscarred uterus. Maternal age was significantly associated with placenta previa.

Placenta previa poses a severe threat to both the mother and the unborn child. The most favourable outcome can be achieved by obtaining an accurate diagnosis and practising prudent expectant management. Disastrous outcomes may be avoided by being proactive about potential clinical problems and using conservative care. Considering the high prevalence of placenta previa in women with a scarred uterus, pregnant women with a scarred uterus must undergo improved monitoring and management.
